# Examining Chat GPT with nonwords and machine psycholinguistic techniques

**DOI:** 10.1371/journal.pone.0325612

**Published:** 2025-06-06

**Authors:** Michael S. Vitevitch

**Affiliations:** Spoken Language Laboratory, Department of Speech-Language-Hearing, Sciences & Disorders, University of Kansas, Lawrence, Kansas, USA; Hong Kong Baptist University, HONG KONG

## Abstract

Strings of letters or sounds that lack meaning (i.e., nonwords) have been used in cognitive psychology and psycholinguistics to provide foundational knowledge of human processing and representation, and insights into language-related performance. The present set of studies used the machine psycholinguistic approach (i.e., using nonword stimuli and tasks similar to those used with humans) to gain insight into the performance of Chat GPT in comparison to human performance. In Study 1, Chat GPT was able to provide correct definitions to many extinct words (i.e., real English words that are no longer used). In Study 2 the nonwords were real words in Spanish, and Chat GPT was prompted to provide a word that sounded similar to the nonword. Responses tended to be Spanish words unless the prompt specified that the similar sounding word should be an English word. In Study 3 Chat GPT provided subjective ratings of wordlikeness (and buyability) that correlated with ratings provided by humans, and with the phonotactic probabilities of the nonwords. In Study 4, Chat GPT was prompted to generate a new English word for a novel concept. The results of these studies highlight certain strengths and weaknesses in human and machine performance. Future work should focus on developing AI that complements or extends rather than duplicates or competes with human abilities. The machine psycholinguistic approach may help to discover additional strengths and weaknesses of human and artificial intelligences.

## Introduction

The methodological techniques commonly used in Cognitive Psychology to examine the inner workings of the “black box” that leads to human behavior have been applied in the emerging disciplines of machine psychology [1] and artificial cognition [2–5] to examine the black box-like behavior of artificial intelligence. In the present set of studies methodological techniques commonly used in psycholinguistics were used in an approach that might be described as *machine psycholinguistics* to examine the language-related behavior (as opposed to decision making or problem-solving behaviors) of a widely-used LLM, Chat-GPT. Specifically, various types of nonwords were presented as stimuli, and the responses generated by the LLM were examined in various ways. The goal of the present studies was not to determine if the LLM used representations and processes that were similar to or different from those used by humans to perform the psycholinguistic tasks (because most assuredly they are different). Rather, the goal of the present studies was to examine the ways in which performance differed in LLM and humans in order to highlight the strengths and weaknesses of each type of intelligence. Determining the strengths and weaknesses of each type of intelligence can provide guidance on how LLM/AI might be used in the future to augment and expand human intelligence instead of developing LLM/AI that simply replicates human abilities.

Nonwords have long been used and have played an instrumental role in the cognitive and language sciences. Consider the pioneering research on human memory carried out by Hermann Ebbinghaus in the 1800’s. To examine how quickly and accurately information was encoded into memory, how long that information persisted, and how quickly information that had been forgotten could be relearned, Ebbinghaus developed a methodology that employed specially constructed syllables that contained a vowel placed between two consonants and were devoid of meaning (e.g., kig, naf, sep) [6]. By memorizing lists of such nonwords until he had perfect recall, then assessing his ability to recall the lists of nonwords after varying delays, Ebbinghaus discovered (among other things) the exponential loss of information in what has come to be known as the forgetting curve (which shows that a large amount of information is lost almost immediately, but a smaller amount of information may persist for a significant amount of time). This finding was replicated over a century later [7], and is a fundamental topic in textbooks for classes in Introductory Psychology [8] and Cognitive Psychology [9].

In the language sciences, the wug-test is a well-known example of nonwords being used to test children’s implicit knowledge of English morphology [10]. A classic example of this task (from which the name of the test is derived) is: *This is a wug. Now there is another one. There are two of them. There are two _____.* A child who understands how to produce the plural form of a noun in English (by adding the morpheme -*s*) will respond with “wugs.” The pioneering work in [10] examined a variety of morphological rules in English in children. This work showed (in contrast to the Behaviorist zeitgeist) that children did not acquire language by simply retrieving previously learned examples (otherwise the children would not be able to produce “wugs” in response to the novel presentation of “wug”). This work also demonstrated that research could be performed with children, and laid the foundation for the experimental field of psycholinguistics.

There is also a history of employing nonwords in psycholinguistic research with various types of artificial neural networks (ANN). Specially constructed nonwords are often used to create a lexicon that is well-controlled and manipulated (often in a way that cannot be ethically or feasibly done with humans) for an ANN to learn and be tested on in some way (e.g., [11]). Another well-known use of nonwords in an ANN related to language processing is the demonstration in the TRACE model of speech perception that effects of phonotactics, or the order in which phonological segments occur in a word, could arise from conspiracy effects among words in the lexicon rather than explicitly coded rules of phonotactic sequencing [12]. That is, the presence of several words in the lexicon of the model that start with /tr_/ (such as trip, trick, etc.) and /sl_/ (such as slip, slick, etc.) provides the model with enough information to identify an ambiguous phoneme (something between an /l/ and /r/) as an /r/ when it is in the context of /t*i/ but as an /l/ in the context of /s*i/ without having to encode or search a list of phonotactic rules for English. Given the long history and important ways in which nonwords have been used in the cognitive and language sciences, various types of nonwords were used in the present set of studies along with a machine psycholinguistic approach to examine the language-like performance (not the processes and representations per se) of a widely-used LLM, Chat-GPT.

In Study 1 the LLM was prompted for the definition (i.e., semantic information) of extinct words in English. That is, the stimuli were considered real words in English at one time, but have fallen out of everyday use in contemporary English, and therefore have no meaning to a modern-day speaker of English (i.e., they are now considered nonwords). Variations of this task (i.e., asking for a definition of a nonword) can be seen in the board game *Balderdash!*, and in psycholinguistic investigations of the bouba/kiki effect (where correlations are observed between speech sounds and visual characteristics of objects [13]). In this task the number of correct definitions was the dependent measure. Given that these stimuli are not used in contemporary English a modern-day speaker of English is unlikely to produce a correct definition to any of the items. In the case of Chat-GPT, which has been trained on an extensive collection of text-based materials, definitions of these archaic words might exist somewhere in its “memory.”

In Study 2 the nonwords were real words from a language other than English; in this case the words were from Spanish. The LLM was prompted to respond with an English word that sounded like the nonword stimulus. This task is similar to the phonological associate task that has been used with nonwords in English speakers [14] and with real words in English speakers [15] to assess different dimensions of phonological similarity. The same metric used in previous studies with humans [14–15] was used in the present study to determine the extent to which the response generated by Chat GPT sounded similar to the cue item (i.e., how many phonemes differed between the cue and response).

In Study 3 nonwords were specially constructed to resemble to a greater or lesser extent real words in English. That is, the nonwords varied in phonotactic probability, or the frequency with which phonological segments and sequences of phonological segments occur in words in a language [16]. Chat GPT was prompted to rate on a scale from 1 (corresponding to “Bad English word”) to 7 (corresponding to “Good English word”) the extent to which the nonword sounded like an English word. The ratings provided by Chat GPT were compared to the ratings provided by native speakers of English.

Note that Study 1 examines semantic information (i.e., the meaning of a word), whereas Studies 2 and 3 examine phonological information, or knowledge related to the speech sounds that make up a word. A crucial point to remember is that the training data for Chat GPT is written text, not spoken language. Thus, any phonological information about how a word “sounds” must be derived indirectly. In languages with a shallow orthography like Spanish there is a (nearly) one-to-one mapping between letters and sounds [17]. Thus, it may be relatively easy to determine that the /f/ sound is always made by (and only by) the letter *f*. However, in languages with a deep (or opaque) orthography like English or French there is more variability in the mapping between letters and sounds. Consider for example the /f/ sound in the English words: fig, phone, cuff, and cough (and then contrast the sound made by the -gh- letters in *cough* with the sound not made by those letters in the word *dough*, and the sound made by those letters in the word *ghost*). The variability in the mapping between letters and sounds in languages with deep orthography (like English) may increase the difficulty of extracting phonological information from written input, which may make the psycholinguistic tasks used in Studies 2 and 3 a potential challenge for Chat GPT.

In Study 4, Chat GPT was prompted to create a new English word to refer to a novel concept presented in the prompt. Such nonwords are commonly used for comedic effect [18], but in the present context they enable us to examine how semantic and phonological (and perhaps morphological) information might interact. In all four studies the aim of presenting nonwords to Chat GPT and of analyzing in various ways the output was to assess the abilities and determine the limitations of LLMs using the methods of machine psycholinguistics. Determining how and where the performance of artificial intelligence may exceed the performance of human intelligence could provide researchers with important information that could be used to develop devices that provide augmented and assistive intelligence (AAI) to overcome the cognitive limitations inherent to human intelligence. Such AAI devices would be akin to the Augmented and Assistive Communication (AAC) devices used by individuals with difficulty communicating verbally.

### Study 1: Extinct English words as nonwords

Much like an extinct species or an extinct volcano, words that are no longer commonly used in a language might be described as “extinct words” (the equally descriptive phrase “linguistic fossils” is used in [19] pg. 9). An example of such a word is *upknocking*, which describes a 19th century occupation (i.e., waking people at a pre-determined time in the early morning) that is no longer a form of employment given the widespread availability of alarm clocks [19]. Because such words are not presently used in everyday speech/text, the definitions associated with these sequences of letters are not likely to be known by most human speakers of contemporary English, and can therefore be considered nonwords.

In this study words that have fallen out of common usage in contemporary (American) English were selected from the collection of words found in [19], and presented to ChatGPT with the prompt: *Define <*word* > *. The number of words not defined (e.g., Chat GPT responded that the word “…is not a recognized term in standard English dictionaries…”), and the number of words defined correctly (i.e., the definition matched the definition in [19]) were assessed. The content of definitions that were provided for some words was examined further. In these cases, Chat GPT provided a definition, but the definition did not match the definition in [19]. Google Books Ngram Viewer [20] was also used to determine the year of peak usage for each extinct word.

### Method

A sample of words was selected from a dictionary of extinct words [19]. The words included in [19] do not constitute an exhaustive list of such words, but were subjectively selected due to their “eccentric phonic essence” (i.e., they sounded funny), and because they captured “…humorous aspects of Old World life” ( [19]; pg. 9). Further, technical terms and non-English foreignisms were excluded from the collection [19].

From the collection of words in [19], 52 items were selected with 2 words starting with each of the 26 letters of the English alphabet. Phrases and hyphenated or monosyllabic words were excluded. Preference was given to words that contained prefixes, suffixes, and base words (i.e., they contained some (pseudo)morphological complexity), but not words that contained transparent morphology (e.g., even if one has never heard the word *biblioklept* it might be easily inferred that it refers to someone who steals books), or extinct words that were related to a word that remained in common usage (e.g., peccable/impeccable or ruly/unruly). Words that resembled a typographical or spelling error were excluded (e.g., bridlegged). When possible, phonotactic and orthographic regularity was preferred, and one word in each letter pair had a singleton in the onset and a consonant cluster in the onset. See the Supporting Information for the words that were selected from [19]. From June 3–7, 2024, varying numbers of the 52 words were pseudo-randomly selected and presented to Chat GPT 4o with the prompt: *Define <*word* > .*

### Results

Of the 52 extinct words that were presented, Chat GPT provided a correct definition (i.e., matching the definition in [19], or the definition from [21]) for 36 items (69.2%). For 11 items (21.2%), Chat GPT was not able to provide a definition, and instead responded with:

*[cue word] does not appear to be a recognized word in standard English dictionaries or common usage. It might be a misspelling, a very obscure term, or specific to a certain dialect or context that is not widely documented. If you have more context or details about where you encountered the term, I could help you further in trying to determine its meaning.*

For the remaining 5 items (9.6%), Chat GPT provided a definition, but that definition did not match the definition in [19 or 21]. Inspection of the definitions that fell into this category shows that 3 of the definitions were connected to “foreign” words or concepts. For example, wangary (soft and flabby meat; [19]) is (correctly) identified by Chat GPT as a town in South Australia. The word lagam (goods which are sunk with a buoy attached to facilitate later recovery; [19]) is identified by Chat GPT as a form of the Tagalog verb “lagom” (meaning to summarize). The word zoldering (an opprobrious epithet; [19]) is identified by Chat GPT as being similar to the Dutch word “zolder” (“attic” in English), and therefore could relate to constructing or renovating an attic, or be a regional term for the attic space itself.

For the remaining 2 items, Chat GPT provided a definition, but that definition did not match the definition in [19 or 21]. The word flothery (slovenly, but attempting to be fine and showy; [19]) was defined by Chat GPT as “…light, airy, or fluffy…soft and delicate in texture or appearance, akin to fluff or froth.” The word tantrels (idle, unemployed people; [19]) was defined by Chat GPT as “…youngsters who are playful in a troublesome or disorderly manner…” These definitions might be described as a form of hallucination, or output from an LLM that is nonsensical or inaccurate [22,23].

Google Books Ngram Viewer (July 2024 dataset; [20]), which contains words and phrases from books published from 1800–2022, was used to determine the year in which the 52 words used in the present study experienced peak frequency of usage. Data were available for 40 of the 52 words. An ANOVA (using [24]) revealed that there was no difference in the year of peak frequency of usage (*F* (2,37) = 0.42, *p* = .66) for the words that were defined correctly (*n* = 30, *mean* = 1855.33 CE, *sd* = 47.66), the words that Chat GPT could not define (*n* = 8, *mean* = 1868.5 CE, *sd* = 52.80), and the words that Chat GPT defined incorrectly (*n* = 2, *mean* = 1879.00 CE, *sd* = 2.83).

### Discussion

In the present study extinct words were presented to Chat GPT with the prompt to define the word. Despite the words not being frequently used in English for approximately 165 years, Chat GPT produced a correct definition to 69.2% of the words. Given the amount of time since these letter sequences were frequently used to convey meaning in English (and the inclusion of such words in a curated collection of extinct words; [19]), it is unlikely that a human speaker of contemporary English would attain a level of accuracy comparable to Chat GPT if asked to define the same extinct words. Rather, a speaker of contemporary English would most likely consider all of these letter sequences to be devoid of meaning, and therefore nonwords, which was indicated for 21.2% of the words by the response from Chat GPT that the word “…*does not appear to be a recognized word in standard English dictionaries or common usage.”* For the remaining 9.6% of the words, Chat GPT provided definitions that were “hallucinations.” That is, the definitions were not consistent with the definitions found in established sources, and were instead nonsensical or inaccurate [22,23].

The results of the present study demonstrate one way that artificial intelligence can be used to augment or assist in some way human intelligence. Consider the human cognitive process of collective memory, or the body of knowledge that we know (or think we know) about the past [25]. Previous studies have shown that collective memory for US Presidents is limited to the 9–10 presidents that served most recently in one’s lifetime [26]. The 9–10 presidents recalled most accurately by students tested in 1974 differed from the 9–10 presidents recalled most accurately by students tested in 1991 (and the same for students tested in 2009 [26]). Beyond that number of presidents, recall performance was significantly reduced in all students, regardless of when they were tested (see [26] for additional caveats).

If we consider “words in the English language” to be a body of knowledge that we (think we) know, then we should expect that over time some words from that body of knowledge will be lost from our collective memory (indeed, enough of them to fill a book; [19]) just as certain US presidents were lost from collective memory in the study by [26]. A well-designed/well-trained AI could enable a modern-day English speaker to reach beyond the limits of their collective lexical memory to correctly identify a letter sequence as a meaningful English word (even if it is a word that is no longer in common use), as Chat GPT did with 69.2% of the extinct words in the present study. Such a cognitive tool could also make a modern-day English speaker aware of other changes to the language that may have taken generations to occur [27], further extending knowledge about the past for a modern-day English speaker.

### Study 2: Words from another language as nonwords

Whereas Study 1 examined meanings (i.e., semantic information) that might be associated with letter sequences, the present study examined phonological information, or knowledge of how the letters in a written word sound when spoken aloud. Given that Large Language Models like Chat GPT receive written/text input during training, any knowledge of how a word sounds when spoken aloud must be obtained indirectly. Across languages there is variability in the depth of the orthography, or the consistency with which letters or sequences of letters map to the phonemes in that language (i.e., [17]). However, within a language, even for a language with a relatively deep orthography like English, there are some regularities and patterns (as well as exceptions) that might be observed and potentially exploited [28]. The present study used techniques from machine psycholinguistics to examine how Chat GPT might use the phonological information that may have been extracted from the texts that were used as input during training.

Previous studies of LLMs used several language-related tasks—grapheme-to-phoneme conversion, syllable counting, and generating a word that rhymes with the cue—commonly used to assess engineered systems that perform natural language processing, text-to-speech synthesis, and automatic speech recognition systems [29]. The same tasks have also been shown to predict success in learning to read in human children, and have been used by speech-language pathologists and other professionals to screen for potential language or reading disorders [30]. In the work by [29], these tasks were used to assess the performance of six different LLMs. It was found that no single model excelled at all three tasks. Rather, one model might have done well in one task, but quite poorly in another. Further, in the grapheme-to-phoneme conversion task, several of the models performed at a level that approached human performance in the task. However, in the syllable counting and rhyme generation tasks, all of the models lagged significantly behind the level of performance observed in humans.

In the present study the LLM was prompted to respond with a word that sounded like the nonword stimulus. A similar task has been used with human speakers of English using real words [15] or English-like nonwords [14] as the prompt. In the present study the nonwords were actually words from another language (i.e., Spanish). However, the same metric used in previous studies with humans [14–15] was used in the present study to determine how similar the response generated by Chat GPT sounded to the cue item.

### Method

Twenty bi-syllabic Spanish words (10 had masculine gender, ending in -o; 10 had feminine gender, ending in -a) were selected from a list of words previously used in [31]. Spanish words with 2 syllables were selected because an analysis reported in [32] found that 90% of Spanish words had two or three syllables, and 80% of English words had one or two syllables (see also [33]). Thus, two syllable words represented a region of overlap for the two languages, increasing the likelihood that the Spanish words would still be somewhat word-like in English (even though the letter sequences are devoid of meaning in English).

Although the real words in Spanish were considered nonwords in English, each Spanish word was phonologically similar to at least one English word as determined by computational analysis [16]. Phonological similarity of the Spanish and English words was defined as the phonological transcription of the Spanish words differing by a single phoneme (via addition, deletion or substitution) from the phonological transcription of a real word in English. From 13–15 August 2024 the Spanish words were pseudo-randomly presented to Chat GPT 4o, first with the prompt *Give me a word that sounds like <word > ,* and, after all 20 items had been presented, a second time (in the same order) with the prompt *Give me an English word that sounds like <word > *. The second round of stimulus presentation and the addition of the word “English” in the prompt occurred because after 3 trials Chat GPT 4o responded to the first prompt with Spanish rather than English words.

### Results

The responses provided by Chat GPT to the nonwords/Spanish words used as stimuli in Study 2 are presented in Table 1. The nonwords are listed in the table in the pseudo-random order in which they were initially presented. English translations are provided in parentheses.

**Table 1:** Responses from Chat GPT from the nonword stimuli in Study 2.

Spanish word (English translation)	Response 1 “*…a word…*”	Response 2 “*…an English word…*”
boda (wedding)	quota	quota
lana (wool)	banana	llama
liga (league)	legal	league
mina (mine)	tina (vat)	meaner
pata (leg)	data (data)	pasta
pena (shame)	tena (have)	pen
beso (kiss)	peso (weight)	vessel
lago (lake)	mango (mango)	logo
lodo (mud)	rodo (rolled)	load
mito (myth)	rito (rite)	meat-o
pelo (hair)	cielo (sky)	pillow
piso (floor)	viso (sheen)	pizzazz
dato (fact)	gato (cat)	dato
foso (grave)	oso (bear)	faux
tela (fabric)	vela (candle)	teller
tubo (pipe)	rubo*	combo
fila (line)	silla (chair)	file
gota (drop)	bota (boot)	goat
gato (cat)	rato (a while)	gato
nata (cream)	nada (nothing)	not a

Note: The nonwords are listed in the order in which they were presented. *rubo is not a word used in contemporary Spanish, but it is similar to rubor (blush) and rubí (ruby).

The same order of presentation was used when presenting the nonwords with the second prompt. Note that after 3 trials using the first prompt (*Give me a word that sounds like <word>*) Chat GPT produced a Spanish word as a response. The one exception during the first round of presentation was the response rubo to the stimulus item tubo (pipe). Although rubo is not a word used in contemporary Spanish, it is a word [I steal] in Italian, another Romance language, and in Galician, another language spoken in Spain ([steal]). Further, rubo is similar to the Spanish words rubor (blush) and rubí (ruby).

In the presentation of the second prompt (*Give me an English word that sounds like <word>*), Chat GPT responded with real English words on most trials. The exceptions included 2 responses that might be described as short phrases (in response to mito and nata), and 2 instances in which the stimulus item was simply repeated in the response (dato and gato). In the case of gato, additional information was included in the response:

*An English word that sounds like “gato” is “gato” (in the context of the Spanish word used in English conversation). A close match would be “gato” when pronounced similarly to “gateau” (a French word for cake).*

The same metric used in previous studies with humans [14–15] was used to determine “how similar” the response generated by Chat GPT sounded to the cue item (i.e., how many phonemes differed between the cue and response). For the responses to the first prompt 85% (17 of 20) differed from the stimulus word by a single phoneme, and 15% (3 of 20) differed from the stimulus word by more than 1 phoneme. The status of the response (e.g., English word, Spanish word, nonword, etc.) was not considered in this assessment. Further, the type of phoneme change (e.g., addition, deletion, or substitution) was not considered in this assessment.

For the responses to the second prompt, 20% (4 of 20) of the responses were phonologically identical to the stimulus word (i.e., 0 phonemes differed), either because the stimulus word was simply repeated (in the case of gato and dato), or because the English “phrase” that was provided as a response was phonologically identical to the Spanish word/nonword stimulus (in the case of mito/“meat-o” and nata/ “not a”). Further, 45% (9 of 20) of the responses differed by a single phoneme, and 35% (7 of 20) differed from the stimulus word by more than 1 phoneme. Again, the status of the response (e.g., English word, Spanish word, nonword, phrase, etc.) was not considered in this assessment, nor was the type of phoneme change (e.g., addition, deletion, or substitution).

### Discussion

In Study 2 Chat GPT was prompted to provide a word that sounded similar to a sequence of letters provided as input. Although the letter sequences presented as the stimulus were real words in Spanish, the letter/phoneme sequences were devoid of meaning in English, making them English-like nonwords.

In the first part of this study, the prompt “*Give me a word that sounds like <word>*” was used. For the first 3 trails, Chat GPT provided an English word as a response to the nonword, but in the subsequent trials it provided (in most cases) a Spanish word that was phonologically similar to the stimulus item. Given that Chat GPT is trained on texts from a number of languages, including Spanish, it is perhaps not surprising that similar sounding Spanish words were presented as responses to the English nonwords (that were real words in Spanish). Such responses are technically correct; the response (in most cases) was indeed a word that sounded similar to the stimulus word. The response was just not an English word like the rest of the words that appeared in the prompt.

In the second part of this study, the same nonwords/Spanish words were presented again to Chat GPT using the more specific prompt “*Give me an English word that sounds like <word>.*” This resulted in most of the responses being English words that differed from the stimulus word by a single phoneme (e.g., added, deleted, or substituted), similar to the behavior of human participants in a comparable psycholinguistic task with either real words [15] or nonwords [14] as stimuli. (The responses provided in the first part of the study also tended to differ from the stimulus by a single phoneme/letter.) Although a computational analysis confirmed that each of the stimulus items had one or more English words that differed from it by the addition, deletion, or substitution of a single phoneme [16], a small percentage of the responses differed from the stimulus item by more than a single phoneme. A few of the responses were also repetitions of the stimulus item.

Previous work examining the ability of LLMs to generate a word that rhymes with the cue found that model performance lagged significantly behind the level of performance observed in humans [29]. In the present study, however, performance of the LLM in a different phonological task was comparable to human performance. Recall that words that rhyme must sound similar at the end of the word (e.g., light and right), whereas the present task left open the possibility that phonological similarity could occur anywhere in the word (e.g., light and like). Compared to the rhyme generation task, the additional freedom in responding that the present task allowed for may account for the better performance in the present study compared to previous studies looking at phonology in LLMs [29].

Although the task used in the present study may have been more flexible than the rhyme generation task used in previous studies [29], it is interesting that other ways to define phonologically similar (e.g., the word cat is embedded in the word catalog) were not observed in the Chat GPT responses. Different dimensions of phonological similarity have been observed in various psycholinguistic tasks with humans [15]. It is unclear what additional modifications to a prompt provided to Chat GPT would be required to elicit similar behaviors in the LLM.

Another behavior of Chat GPT in the present study is worthy of discussion, namely providing Spanish responses to English prompts as observed in the first part of this study. This language switching behavior is not unique to the present study, as indicated by reports from other users of Chat GPT in the OpenAI Developer Forum [34]. On the surface this language switching behavior in Chat GPT may resemble the phenomenon of code-switching in humans who know more than one language [35]. However, providing a response in a language that differs from the language used in the rest of the prompt is on some level inconsistent with (though not an egregious violation of) the unwritten and implicit expectations of human interlocutors to converse in a language(s) that is shared by both speakers. Although LLMs might be able to detect various patterns from the written input they are trained on, including the sounds that letters make in several languages, LLMs are not able to extract “rules” that are not written anywhere. These “rules” include the expectations and contextual knowledge shared by human interlocutors that allow them to communicate without language (e.g., a nod of the head), with non-linguistic noises (e.g., a grunt), using vague or context-dependent terms (e.g., “This one, not that one.”) or minimal linguistic input rather than elaborate, explicit, and engineered “prompts” during interactions.

Additional studies using the machine psycholinguistic approach and simple tasks such as the task employed in the present study may identify more domains, tasks, and abilities related to language and its flexible use that differ between LLMs and humans. Some developers may seek to reduce those gaps in performance to create LLMs that perform as well as or better than humans on language-related tasks. An alternative approach is to harness the differences between LLMs and humans to allow LLMs to complement the cognitive limitations of humans (as described in Study 1) or to allow humans to complement the inflexibility and lack of knowledge of unwritten rules and expectations in LLMs (as in the present study) to enable both entities to cooperatively produce a behavior that neither would be capable of by themselves.

### Study 3: Nonwords varying in phonotactic probability

Study 2 and other work on LLMs [29] directly examined phonological similarity of (non)word stimuli to English words. In the present study, specially constructed nonwords were used to examine meta-phonological information about English words. That is, instead of generating an English word that was phonologically similar to the stimulus, Chat GPT was prompted to “subjectively” rate using a 7-point scale the extent to which specially constructed nonwords that varied in phonotactic information sounded like real English words.

Phonotactic information refers to the rules that govern which speech sounds are used in a language, and how those sounds can be arranged to form words in a language. For example, the “ng” sound is legal at the end of English words (as in the words *ring* and *sing*), but it cannot begin a word in English (however, in Albanian it is legal to start a word with that sound). Looking within a language at only the legal sounds and sequences of sounds, one sees that those sounds and sequences occur with varying frequency. This information is referred to as phonotactic probability [36], and it influences the ability of infants to distinguish between speech in their native language versus another language [37], assists children in acquiring new words [38], is used by infants [39] and adults [40] to segment words from fluent speech, and by adults to produce [41] and recognize spoken words [36].

Phonotactic information not only influences several language-related processes, but it has also been shown to influence other—seemingly unrelated—cognitive processes, such as deciding how much money one is willing to invest in a company that has a name that is easy or difficult to pronounce [42]. In another study human participants who were native speakers of English were asked to rate the wordlikeness and buyability of nonwords varying in phonotactic probability. Nonword stimuli that were rated as being more word-like were also rated as being more likely to be purchased if they were the name of a new product [43]. As noted in [43], brand names are similar to real words and proper names in that they all have a lexeme (i.e., the name) and a lemma (i.e., the sematic information or referent), but they differ from real words and proper names in that they also signal social status and may influence self-esteem. Thus, phonological information may affect more than just language-related processes [44].

The present study used the same nonword stimuli varying in phonotactic probability that were used in [43]. As in [43], Chat GPT was prompted to rate on a scale from 1 (corresponding to “Bad English word”) to 7 (corresponding to “Good English word”) the extent to which the nonword sounded like an English word, and then rate on a scale from 1 (corresponding to “least likely to buy”) to 7 (corresponding to “most likely to buy”) the likelihood of buying a product named <nonword > . The ratings provided by Chat GPT in both tasks were compared to the ratings provided by native speakers of English in [43].

### Method

The nonwords used in the present study were the same 60 consonant-vowel-consonant, monosyllabic nonwords used previously in [43]. Phonotactic probabilities for the stimuli were calculated using the Phonotactic Probability Calculator [16]. In [43], nineteen native English speakers with no reported speech, language, or hearing disorders provided ratings to the nonwords. Those ratings were compared to the ratings provided by Chat GPT in the present study.

In the previous studies with humans, the stimuli were presented auditorily, and participants used a computer keyboard or response box to enter their numerical rating. Because Chat GPT does not directly process auditory input (i.e., spoken input is first converted to text), the phonological transcriptions of the nonwords were converted to orthographic representations using the English Sublexical Toolkit [28] to ensure consistent mapping between spellings and sounds. Spellings were adjusted until each item had 100% spelling-sound mappings as per the English Sublexical Toolkit [28].

From 7–12 August 2024 the nonwords were pseudo-randomly presented to Chat GPT 4o, first with the prompt: *On a scale from 1 (corresponding to “Bad English word”) to 7 (corresponding to “Good English word”) how well does <nonword> sound like an English word?* After all 60 items had been rated for wordlikeness, the nonwords were presented in a different pseudo-random order with the prompt: *On a scale from 1 (corresponding to “least likely to buy”) to 7 (corresponding to “most likely to buy”) how likely would you buy a product named <nonword > *. The same wordlikeness and buyabilty prompts were used in [43].

### Results

As reported in [43], subjective human ratings of wordlikeness correlated significantly with both objective measures of phonotactic probability: sum of the segments (*r* = .55, *p* < .0001), and sum of the biphones (*r* = .56, *p* < .0001). The subjective human ratings of wordlikeness correlated with the subjective human buyability ratings (*r* = .70, *p* < .0001). The subjective human ratings of buyability correlated significantly with both objective measures of phonotactic probability: sum of the segments (*r* = .51, *p* < .0001), and sum of the biphones (*r* = .48, *p* < .0001).

In the present study, the same analyses (using [24]) were performed with the responses produced by Chat GPT. Ratings of wordlikeness from Chat GPT correlated significantly with both objective measures of phonotactic probability: sum of the segments (*r* = .35, *p* < .0001), and sum of the biphones (*r* = .35, *p* < .0001). The ratings of wordlikeness from Chat GPT correlated with the buyability ratings from Chat GPT (*r* = .68, *p* < .0001). The ratings of buyability from Chat GPT correlated significantly with both objective measures of phonotactic probability: sum of the segments (*r* = .51, *p* < .0001), and sum of the biphones (*r* = .50, *p* < .0001).

Because the stimuli presented to Chat CPT were converted to orthographic representations, the same analyses were repeated conditioned on the number of letters in each nonword, reading consistency (*p*(P|G) from [28]), and spelling consistency (*p*(G|P) from [28]). Again, ratings of wordlikeness from Chat GPT correlated significantly with both objective measures of phonotactic probability: sum of the segments (*r* = .43, *p* < .0001), and sum of the biphones (*r* = .42, *p* < .0001). The ratings of wordlikeness from Chat GPT correlated with the buyability ratings from Chat GPT (*r* = .68, *p* < .0001). The ratings of buyability from Chat GPT correlated significantly with both objective measures of phonotactic probability: sum of the segments (*r* = .48, *p* < .0001), and sum of the biphones (*r* = .45, *p* < .0001).

Paired samples t-tests were used to compare the wordlikeness ratings obtained from humans to the wordlikeness ratings obtained from Chat GPT, and the buyability ratings obtained from humans to the buyability ratings obtained from Chat GPT. For the wordlikeness ratings, Chat GPT produced significantly higher ratings (*mean* = 3.94; *sd* = 1.32) than humans (*mean* = 3.53; *sd* = 0.76; *t* (59) = −2.78, *p* < .01). Similarly, for the buyability ratings, Chat GPT produced significantly higher ratings (*mean* = 3.88; *sd* = 1.06) than humans (*mean* = 3.28; *sd* = 0.63; *t* (59) = −5.06, *p* < .0001).

### Discussion

In the present study, a cognitive/psycholinguistic task used with humans in [43] was used with Chat GPT. That is, Chat GPT was prompted with specially created nonwords that varied in phonotactic probability, and instructed to use a 7-point scale to rate how much the stimulus resembled English words and how likely one would buy a product with the name of the nonword. Despite the nonwords being transformed to an orthographic form (i.e., a string of letters) in order to present them to Chat GPT, the performance of the LLM resembled the performance of humans in both rating tasks. That is, the “subjective” ratings of wordlikeness correlated with two measures of phonotactic probability (i.e., the sum of the segments, and the sum of the sequences of segments), the ratings of buyability correlated with two measures of phonotactic probability, and the ratings of wordlikeness correlated with the ratings of buyability. These relationships remained even when the correlations were conditioned on the number of letters in each nonword, and two measures of the consistency in mapping letters and sounds.

The only difference between the ratings provided by humans and by Chat GPT was that the wordlikeness ratings from Chat GPT tended to be higher than those provided by humans. This difference may be due to humans using (on average) a more restricted range of the rating scale (minimum mean rating = 2.32; maximum mean rating = 5.47) compared to the range used by Chat GPT (minimum rating = 2; maximum rating = 7; *N.B*., this may also be an artifact of sampling and statistical measurement), or to humans using factors not used by Chat GPT in assigning their ratings (e.g., “eccentric phonic essence”), or to any number of unmeasured characteristics in the present study.

The ability of an LLM to perform like human language users in assessing meta-phonological information (i.e., how word-like a stimulus sounds) despite receiving only text as input during training may appear at first to be an impressive accomplishment. However, even languages like English with deep orthographies [17] have consistencies and regularities in the mappings between phonemes and letters that can be described probabilistically and potentially exploited [28].

There are many other statistical regularities that have been observed in human languages. One example is Menzerath’s law, which states that an increase in a particular linguistic unit is associated with a reduction in the size of its constituents. Thus, a long sentence (as measured by the number of clauses) will tend to have shorter clauses. Similarly, a long word (as measured by the number of syllables) will tend to be comprised of small or simple syllables (such as CVs or Vs). The relationship between unit and constituent size has also been observed in music and genomes [45]. There are numerous statistical regularities in human languages observed by [33], such as the correlation between word-length and word frequency (i.e., long words tend to occur less often in the language). Also, words that occur often in the language tend to be phonologically or orthographically similar to many other words in the language (e.g., [46]). Given that rodents can be trained to exploit certain regularities in human speech to distinguish between phoneme contrasts in English [47], the ability of a machine that is trained to discover and exploit the numerous regularities found in human languages now appears less impressive.

The results of the present study using the machine psycholinguistic approach show that Chat GPT can discover and exploit regularities in English words to perform a nonword rating task at a level comparable to humans. Using AI to discover these and other regularities in human languages could prove useful to researchers developing natural language processing (NLP) applications and augmented and alternative communication (AAC) devices that enable humans to accomplish more than they would be able to without those applications or devices.

Note, however that the regularities found in English words described above relate to the phonological or orthographic characteristics of the words. There are, of course, regularities in human languages that relate to the semantic characteristics of words, such as the observation that words that occur more often in the language tend to have many meanings [33; 48]. Therefore, in Study 4 we consider the performance of Chat GPT on a semantic task.

### Study 4: Create a new English word for a novel concept

Although some previous work found similarities in how humans and LLMs respond, other previous studies using the machine psychology/psycholinguistic approach have found important differences in the responses generated by humans and the responses generated by LLM. In a semantic associate task (i.e., What’s the first word that comes to mind when you read the word _____?) human generated associates contained less gender bias and were richer and more varied than the associates generated by an LLM [49]. For example, in response to the prompt dog, humans gave cat and other syntagmatically related associates (e.g., love, bone, furry, leash, loyal), whereas the LLM tended to provide associates that were paradigmatically related (e.g., puppy, canine, animal, pet). Understanding such differences in performance may prove useful in determining how best to use AI to complement human performance in a variety of domains.

In the present study Chat GPT was prompted to create a new English word to refer to a novel concept described in the prompt. This task differs from the task used in Study 1 where a definition was requested for a novel word that was presented in the prompt. The task used in the present study enabled us to examine how various types of linguistic information—phonological (perhaps morphological) and semantic information—might interact and be used in a novel and creative way (for examples of the creative use of language in humans see [50,51]). Google Books Ngram Viewer [20] and dictionary searches were used to confirm the novelty of the new word responses.

### Method

Table 2 shows the prompts used in the present study. The prompts were presented to Chat GPT 4o in the order listed in the table on 19-AUG-2024. The new word that was created is also presented in Table 2.

**Table 2:** The prompts presented to Chat GPT, and the novel word produced as a response.

1	Create a new English word that means the feeling of pleasure in someone else’s misfortune.	Mispleasure (1837)
2	Create a new English word that means taking pride in someone else’s achievement.	Prideify
3	Create a new English word that means the craving for salty food, then sweet food, then salty foods again.	Saltuine
4	Create a new English word that means the erroneous perception that the return trip from a destination is shorter in duration than the initial trip to the destination.	Retrimption
5	Create a new English word that means the process by which a nonword begins to take on meaning.	Lexinize
6	Create a new English word that means anger experienced upon being awoken.	Rousrage
7	Create a new English word that means the phenomenon of greeting another person in a familiar manner, then realizing that the person is not who you thought they were, and only resembles that other person.	Misgreet (1851)
8	Create a new English word that means to trip over one’s own feet.	Stumblop
9	Create a new English word that means the fear of being watched by a platypus.	Platypobia
10	Create a new English word that means a container used to restrain cabbages.	Cabbinet (1873)

Note: Peak year of usage is provided (in parenthesis) for the 3 words that were found in Google Books Ngram Viewer.

Although there is a German word (schadenfreude) for the concept described in prompt 1, there currently is no English word in contemporary usage for that concept. The German word schadenfreude is sometimes used by English speakers, however. Similarly, there is a Yiddish word (naches; typically applied to one’s family) that is close in meaning to the concept described in prompt 2, but there currently is no English word in contemporary usage for that concept.

## Results

Google Books Ngram Viewer [20] and searches of on-line dictionaries were used to confirm the novelty of the responses. Three responses from Chat GPT were found in the Google Books Ngram Viewer (see the years of peak usage listed in parenthesis for each word in Table 2). In all 3 cases the peak year of usage was over 150 years ago, and little to no usage was observed for the terms in more recent eras.

In the searches of on-line dictionaries 2 of the 3 words found in the Ngram Viewer analysis had dictionary entries (mispleasure and misgreet) with definitions that differed from the concept described in the prompt. The third word (cabbinet) found in the Ngram Viewer analysis did not have a dictionary entry, suggesting that cabbinet may have been an early spelling variant of the word cabinet.

One other word (prideify) did appear in on-line searches, but not in the Ngram Viewer analysis nor as a dictionary entry. In this case, “pride-ify” (with the hyphen) was used to refer to changing one’s social media profile picture in some way to show support for Gay Pride month.

## Discussion

In the present study we examined how Chat GPT performed when prompted to create a new English word in response to a novel concept, enabling semantic, phonological, and morphological information in the LLM to be examined. In two cases, sequences of letters were produced as novel words that were in fact extinct English words like those examined in Study 1 (but in the present case whose new meaning differed from the actual meaning of the extinct word). Recall that in Study 1 when Chat GPT was prompted to define extinct English words approximately 90% of the extinct word prompts were defined correctly or flagged as possibly being words that are no longer in use, with the remaining 10% being defined incorrectly. In the present study, Chat GPT tried to redefine (or define incorrectly) two extinct English words. Incorrectly defining extinct words in Study 1 and in the present study may be examples of the LLM “hallucinating,” or producing output that is nonsensical or inaccurate, or that refers to evidence (e.g., scientific journal articles) that does not exist [22,23].

Note, however, that humans also produce inaccurate output, as seen in the occurrence of malapropisms [52], or whole word speech errors that are phonologically but not semantically similar. An example is substituting the word flamingo (a bird) for the intended word flamenco (a type of dance). Just as insight into human language processing is obtained from the analysis of patterns observed in human speech errors [52], similar analyses of error patterns in Chat GPT using the machine psycholinguistic approach might provide researchers with additional insight in to how to best use AI to complement the abilities of human intelligence and language processing abilities.

The remaining responses in the present study involved cases of portmanteau, or the combination of the sounds (or spellings) and meanings of two existing words to form a new word. Familiar examples of this phenomenon in English are the words brunch (breakfast + lunch), and motel (motor + hotel). One example of portmanteau from the present study is the new word rousrage (rouse + rage).

Although some of the responses in the present study contained letter sequences that appear to be English morphemes, the remaining sequences of letters in those novel responses suggest that the response might be better described as additional cases of portmanteau rather than novel creations derived from the principles of English morphology. For example, the response lexinize contains the -ize letter sequence, which could be the -ize morpheme that conveys the process of making or becoming, as in the word fossilize (turning into a fossil). The remaining portion of the response is the letter string lexin, which could be described as a shortened form of the word lexicon (referring to a collection of known words), or it could simply be a spelling error. Another example of a possible morpheme/possible spelling error is the response platypobia. A phobia is an extreme and irrational fear of something, but pobia is not a word or morpheme in English. Further, the etymology of the word platypus renders the meaning flat-footed. Thus, platyphobia would refer to a fear of flat things, not a fear of the Australian mammal.

One response where English morphology might be present is prideify. The -ify morpheme changes a noun into a verb in English, and generally means “to cause to be,” as in solidify (i.e., causing something to become solid). Therefore, prideify could refer to an action or event that causes someone to be filled with pride. However, a different parsing of that letter sequence might yield pri-deify, which not only changes the stress pattern of the word, but might also alter the meaning (pri- means to resemble a saw + deify means to regard something as a god, which could be interpreted as the worship of bread knives). Thus, Chat GPT can in some cases create novel words. However, the quality, validity, and true novelty of those words (and of the errors) requires more extensive and more strenuous evaluation than the assessment provided in the present study. The present analysis is intended only to illustrate how the machine psycholinguistic approach might be used to find tasks or domains in which the performance of AI might complement the performance of humans.

### General discussion

Nonsense, in the form of specially created sequences of letters or sounds that lack semantic content, has played an important role in the cognitive and language sciences as a tool to probe the implicit knowledge that human language users represent in memory as well as the processes used to retrieve those representations. In the present set of studies, nonwords of various kinds were presented to ChatGPT with prompts that resembled the instructions of tasks previously used in psycholinguistic experiments with humans. In some of the studies the responses from ChatGPT were compared to previously collected data from human language users to better understand the performance of ChatGPT in those tasks. Future studies using the machine psycholinguistic approach to understand how the behavior of ChatGPT resembles and differs from the performance of human language users as well as the type of errors each entity makes may lead to advances in the development of LLMs and other forms of AI that enhance rather than replicate human cognitive and linguistic abilities.

#### Limitations of the present work.

Future studies using the machine psycholinguistic approach to examine LLMs and other AI models may also overcome some of the limitations of the present set of studies related to the language that was used, the number of trials in some of the studies, and the use of only one version of Chat GPT (or one type of LLM). Regarding the use of the English language in the present studies, even though nonwords were used in the present set of studies, the nonwords were referenced to real words in English. In Study 1 the nonwords were extinct English words. In Study 2 the nonwords were Spanish words, but the response was supposed to be an English word that sounded similar to the nonword prompt. In Study 3 the nonwords varied in the phonotactic probability of speech sounds found in English words. In Study 4 Chat GPT was prompted to create a new English word. The over-reliance of the cognitive and language sciences on English has been increasingly discussed [53,54]. However, the strengths and weakness of LLMs (discussed in more detail below) are more likely to be related to the computational architecture of the model than the language of the training data, prompts, etc.

Another limitation of the present set of studies relates to the small number of stimuli that were used compared to previous psycholinguistic studies with humans. It is true that a larger number of stimuli and a larger sample size of participants have many benefits for statistical analysis (e.g., representativeness of population, less variability, etc.). However, in the present studies the focus was more on the nature of the responses (and the errors) from the LLM (e.g., X rather than Y was observed), rather than the number of certain responses (e.g., a difference in the number of X compared to Y was observed), and was intended to illustrate how the machine psycholinguistic approach might be useful. The small number of stimuli used in the present studies therefore does not diminish the broader conclusions discussed below.

A final limitation of the present studies is that a single version of Chat GPT (and only that LLM) was used across the different studies. Note that other studies have examined behavior in a range of LLMs, and found qualitatively similar results among the models [29]. Further, despite improvements of various benchmarks in each new version of a given LLM [55], the present studies still revealed unusual behaviors and limitations in the performance of the LLM. The unusual behaviors and limitations of LLMs—as well as how the behaviors of LLMs may complement human behavior—will be discussed further below.

#### Cognitive processing in humans and LLMs.

Some previous work using what is described here as the machine psycholinguistic approach found that LLMs produce ratings for concreteness, valence, and arousal to multi-word expressions that were strongly correlated to the same ratings produced by humans [56]. Correlations between LLM and human ratings of word similarity, contextualized sensorimotor associations, and iconicity judgements have also been found [57]. Similarities in ratings provided by LLMs and humans (as in Study 3 of the present work) may lead one to conclude that LLMs can be used to learn something about human cognition and behavior. Such a conclusion would be a mistake, because even though transformers and artificial neural networks are said to be inspired by how neurons in the human brain work [58], real neurons are more complicated [59,60] and do not work like the “processing units” found in these computational models.

The differences in the underlying architectures of computational neural networks and biological neural networks highlights the fact that LLMs are an engineered machine that is very different from the evolved human brain and mind. Although both are performing what appears on the surface to be the same type of cognitive labor, the means by which those outputs are achieved are as different from each other as the form of transportation performed by the wheels and engine in an automobile and the feet of a human, or the form of flight achieved by the static wings and engine of an airplane and the flapping of wings in a bird or bat. In these instances, transportation over land and flight through the air are achieved to differing levels of success by the different systems. But it would not be realistic to expect a car to go where there are no roads even though human feet might deftly maneuver such terrain, or to expect a bird to fly to the moon even though engineered vehicles have flown to the moon on multiple occasions. Similarly, certain forms of cognitive labor may be better performed by a biological system, whereas other forms of cognitive labor may be better performed by engineered systems. The methods of machine psycholinguistics, machine psychology [1], and artificial cognition [2–5] might be useful in determining which cognitive tasks are better suited to engineered versus biological systems, and how the two different systems might best work together to accomplish a task that neither could by themselves.

Although some previous work found similarities in how humans and LLMs respond, other previous studies using the machine psychology/psycholinguistic approach have found important differences in the responses generated by humans and the responses generated by LLM. Using surveys that examined political orientation, economic preference, judgement, and moral philosophy [61], it was found that Chat GPT 3.5, unlike humans, tended to fixate on an answer despite changes to demographic details in the prompt, or changes in the order of presenting the prompts. It has also been asserted that LLMs can learn languages that are impossible for humans to learn [62] (for evidence to the contrary see [63]). All of these studies—the ones that find similarities and the ones that find differences in how humans and LLMs perform—are important because they allow us to determine the strengths and weaknesses of the two systems [64,65].

Determining the strengths and weaknesses of human perceptual and cognitive systems has been the decades-long research focus of cognitive psychology [66] and cognitive science [67]. As discussed in the context of Study 1, human collective memory has a limited time-frame in which optimal performance is observed [26]. Some other perceptual and cognitive limitations in humans include the limits of short-term memory (i.e., 7 + /-2 chunks [68]; *cf*., [69]), subitization, or the direct perception of a small number of visual [70] or auditory [71] objects before explicit counting must occur, finite computational resources that are allocated via attention [72], as well as various biases that may negatively influence decision making processes [64,65,73].

Despite the weaknesses of human perception and cognition, human perception and cognition also has a number of strengths (especially when compared to AI). For example, humans are quite good at abstracting information from previous experience [74]. But, as observed in Study 2, Chat GPT did not use the abstract information related to conversational interactions found in Grice’s Maxim of relation [75], and instead responded with Spanish words even though most of the words in the prompt were English words. Humans are also good at stretching and warping perceptual representations to emphasize certain features to make categorization more accurate [76], and at stretching and warping lexical representations to use language in creative and humorous ways [50,51,77]. The stretching and warping of perceptual and semantic representations also enables humans to make assessments at several scales or levels of analysis, whereas LLMs tend to provide feedback that is more general in nature [78]. Although the responses from Chat GPT in Study 4 showed some promise of being creative, future collaborations of human cognitive systems and engineered cognitive systems might be more successful if the flexible thinking and creative work is left to the humans.

Current research continues to reveal the limits of engineered cognitive systems, especially related to the ability to learn. For example, most LLMs/artificial neural networks that use gradient decent learning algorithms require thousands of exposures to learn the relationship between input and output patterns. However, humans can learn much more quickly by abstracting general principles from previous instances, through trial and error, and via verbal instruction [74]. Further, humans can learn one task, then quickly switch to learning another task (known as plasticity) without catastrophic forgetting of the previous task, but contemporary artificial neural networks (which underlie LLMs) are not able to do so [79]. It is not clear if alternative network architectures or learning algorithms, such as adaptive resonance theory [80], are able to scale-up to match the size of contemporary deep-learning networks and overcome these shortcomings.

Engineered systems that replicate in some way biological systems might help improve the performance of those engineered systems (e.g., [81]). However, to improve the performance of human cognitive processing, mimicry of human cognition in an engineered system may not be sufficient. A different approach might be required, namely, to engineer cognitive systems that complement human cognitive abilities by supplementing or overcoming in some way the limits of human processing (some of which were discussed above). An example of an engineered cognitive system overcoming the human limits of collective memory was observed in Study 1 when Chat GPT correctly identified some (but not all) of the nonwords as extinct words that are no longer used in contemporary English. The resulting engineered systems may more closely resemble automobiles and airplanes than feet and bird wings, but the behaviors such engineered systems exhibit would extend human cognition much like automobiles and airplanes have extended human mobility beyond the limits of feet and wings.

Chat GPT and other AI agents could also extend human cognition when information stored in human semantic memory is not retrieved for some reason. An example of semantic information not being retrieved even though it is known to the speaker occurs in the tip of the tongue (ToT) phenomenon [82]. When a human speaker is in the tip of the tongue state information related to the meaning, gender, or syntactic class of the word may be accessible, but not the complete phonological form of the word. Failures to retrieve words from the lexicon naturally occur for human speakers, with the number of such failures typically increasing with age [83]. Similar retrieval failures occur more frequently for individuals with the word finding problem known as aphasia, which often occurs following a stroke or other damage to language-related parts of the brain [84]. As reported in [85], younger (~2% of the trials) and older adults (~3.5% of the trials) occasionally failed to retrieve a word (i.e., experienced the tip of the tongue state for a set of monosyllabic English words). However, (in a study not reported here) Chat GPT never failed to retrieve the correct word in response to the prompts from [85] that were used to elicit the tip of the tongue state in humans, demonstrating one way (i.e., to retrieve information that is inaccessible during a ToT state, or to someone with aphasia) that LLM could be used to extend or supplement human cognition.

Future efforts to extend or supplement human cognition with AI should proceed with caution. The United Nations Educational, Scientific and Cultural Organization (UNESCO) has provided guidance on several ethical issues related to the increasing use of AI, including equity in access, maintaining privacy of individual data, and prevention of harm especially in medical or health settings [86]. Although extensive in scope, the UNESCO recommendations do not address the issue of whether AI efforts should attempt to duplicate human cognitive abilities or extend human cognitive abilities. Given that part of the mission of UNESCO is to “…foster science and technology in the service of humanity…” [87], the recommendations on AI, especially those related to how AI might be used in the service of humanity, may need to be revised, especially with the emergence of open-source LLMs that allow for transparency, reproducibility, and adherence to data protection standards compared to closed-source LLMs [88].

Concern about the data used to train LLMs is not purely academic. LLMs are trained on various sources of written information, but as seen in Study 2, there are many rules and expectations (such as when to switch from using one language to another) that are not explicitly documented anywhere. Using the machine psycholinguistic approach might help researchers better understand how the sum of human data used to train LLM and the common errors that humans make can be used to best augment the cognitive labor of an individual in a certain task. Just as humans and dogs work together in certain contexts (e.g., herding of farm animals and security applications such as bomb detection), perhaps humans and a well-trained AI could work together in certain contexts to accomplish more than either could alone.

## Supporting information

S1 File Supporting_Information_file.docx The list of extinct words used in Study 1. (DOCX)
