# Supplementary material for: Examining Chat GPT with nonwords and machine psycholinguistic techniques
Source: PLoS One. 2025 Jun 6;20(6):e0325612. doi: 10.1371/journal.pone.0325612 (PMC12143520; doi:10.1371/journal.pone.0325612)
Supplement: S1 File — (DOCX) [file pone.0325612.s001.docx]

**Supporting Information**

Extinct Words used in Study 1

The words used in Study 1 were sampled from [ref 19]. The peak year of usage for each word is in parentheses (if the word was found in Google Books Ngram Viewer [ref 20]).

accubitus (1840)

aflunters

bibitory (1835)

blepharon (1857)

clointer

conskite (1822)

davering (1835)

deosculation (1880)

empasm (1838)

encraty (1965)

farded (1800)

flothery (1877)

gloppened (1874)

gowpen (1829)

haspenald (1849)

huchet (1829)

incalescence (1819)

insufflation (still in use in 2022 in medical, religious and pharmaceutical contexts)

jampher (1895)

jussel (1812)

kimbly (1881)

knotchel (1820)

lagam (1881)

linctus (1807)

maffle (1803)

megrim (1875)

nerled (1904)

nobbler (1945)

oblat (1816)

ostentiferous

palliard (1812)

pulicosity

quanked (1877)

quignogs

raddlings (1819)

rowel (1800)

saloop (1825)

skilly (1864)

tantrels

thrunched

ugsumness

urtication (1857)

vigerage

viridate (1859)

walapang

wangary

xantippe (1915)

xyster (1897)

yaffle (1894)

yeepsen (1862)

zoldering

zwodder (1875)
